# Insights from 20 years of the Molecule of the Month

**DOI:** 10.1002/bmb.21360

**Published:** 2020-06-17

**Authors:** David S. Goodsell, Christine Zardecki, Helen M. Berman, Stephen K. Burley

**Affiliations:** ^1^ Research Collaboratory for Structural Bioinformatics Protein Data Bank, Rutgers The State University of New Jersey Piscataway New Jersey USA; ^2^ Institute for Quantitative Biomedicine, Rutgers The State University of New Jersey Piscataway New Jersey USA; ^3^ Department of Integrative Structural and Computational Biology The Scripps Research Institute La Jolla California USA; ^4^ Department of Chemistry and Chemical Biology The State University of New Jersey Piscataway New Jersey USA; ^5^ Research Collaboratory for Structural Bioinformatics Protein Data Bank, San Diego Supercomputer Center University of California, San Diego La Jolla California USA; ^6^ Rutgers Cancer Institute of New Jersey, Rutgers The State University of New Jersey New Brunswick New Jersey USA; ^7^ Department of Biological Sciences University of Southern California California Los Angeles USA

**Keywords:** biochemistry, Protein Data Bank, protein structure and function, structural biology

## Abstract

For 20 years, Molecule of the Month articles have highlighted the functional stories of 3D structures found in the Protein Data Bank (PDB). The PDB is the primary archive of atomic structures of biological molecules, currently providing open access to more than 150,000 structures studied by researchers around the world. The wealth of knowledge embodied in this resource is remarkable, with structures that allow exploration of nearly any biomolecular topic, including the basic science of genetic mechanisms, mechanisms of photosynthesis and bioenergetics, and central biomedical topics like cancer therapy and the fight against infectious disease. The central motivation behind the Molecule of the Month is to provide a user‐friendly introduction to this rich body of data, charting a path for users to get started with finding and exploring the many available structures. The Molecule of the Month and related materials are updated regularly at the education portal PDB‐101 (http://pdb101.rcsb.org/), offering an ongoing resource for molecular biology educators and students around the world.

## 1 HISTORY

The Molecule of the Month series^1^ was launched January 2000 on the homepage of the newly established RCSB Protein Data Bank (RCSB PDB^2^) portal to the PDB archive. With more than 11,000 structures at the time, the PDB was undergoing a transformative change from an archive of structural biology data to an essential interdisciplinary tool for research, education, and outreach. The RCSB PDB wanted to support its growing user community and expand the utility of the PDB as an educational resource, so a plan was conceived to use a growing collection of structure‐based molecular stories developed to test prototypes of BioEditor,^3^ software for creating annotated 3D molecular visualizations similar to JSmol,^4^ or Kinemages.^5^ Articles in the first year highlighted foundational stories of structural biology, including features on myoglobin, bacteriophage phiX174, DNA polymerase, collagen, cytochrome c oxidase, HIV‐1 protease, nucleosomes, restriction enzymes, lysozyme, ribosomal subunits, rubisco, and pepsin. Interest in the feature was immediate. By March 2000, the column was highlighted by *Science* as a “NetWatch Hot Pick”,^6^ and later a “Website of Note” by *BAMBEd* in 2006.^7^

**FIGURE 1:**
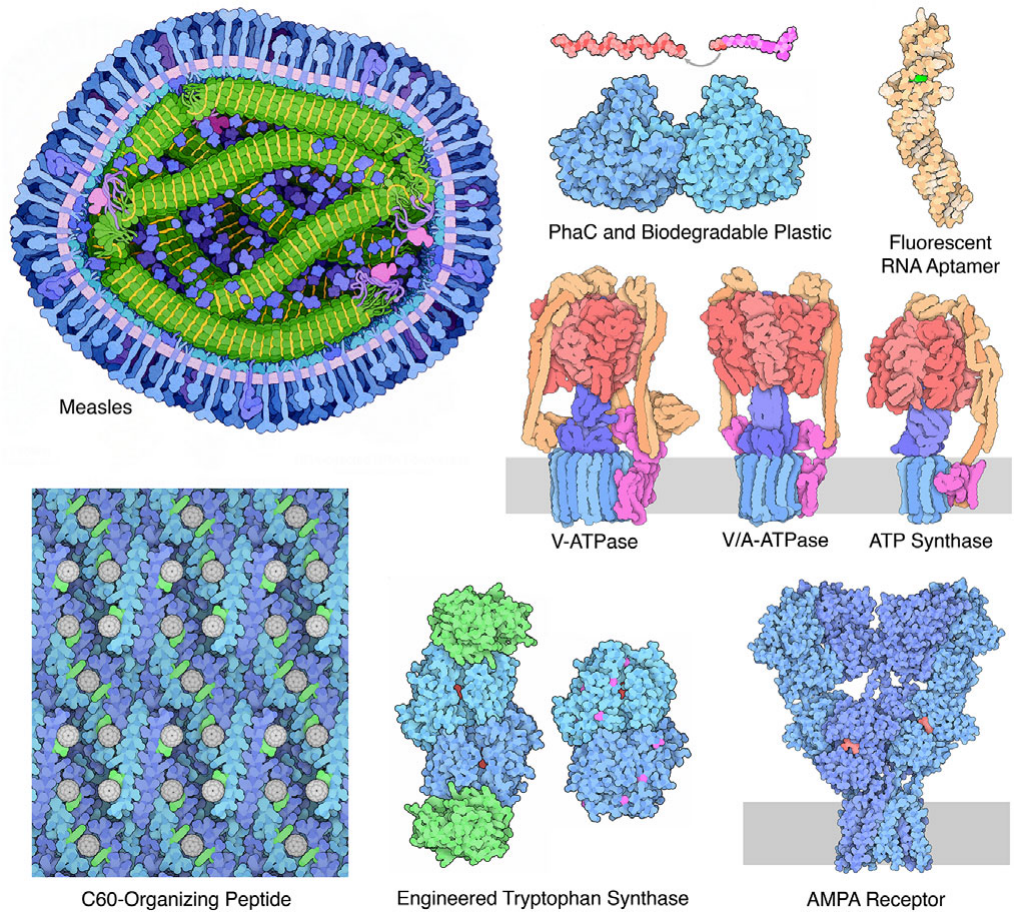
Recent Molecule of the Month articles, with topics selected with different goals. Measles is a topical subject in the news; PhaC and biodegradable plastic relates to the environment; fluorescent RNA aptamers were a reader's choice topic; the rotary ATPases highlighted new cryoEM results and have a central function in energy production; C60‐organizing peptide is an exciting success of nanotechnology; engineered tryptophan synthase relates to the 2018 Nobel Prize in chemistry and is an application of biotechnology; and AMPA receptors were featured to support the upcoming PDB‐101 yearly theme on “Drugs and the Brain.” A full listing of all entries is available at http://pdb101.rcsb.org/motm/motm‐by‐title [Color figure can be viewed at wileyonlinelibrary.com]

Each Molecule of the Month article introduces the structure and function of a biological macromolecule and discusses its relevance to human health, welfare, and disease (Figure 1). The columns are built around characteristic illustrations of the molecules, created directly from atomic coordinates from the PDB archive, using a nonphotorealistic rendering technique consisting of flat, pastel colors with black outlines. Building on these illustrations, the visual identity of the column has evolved over the years. Early illustrations used traditional atomic coloring, and the columns included suggestions for generating similar pictures in RasMol.^8^ Later editions moved to a more function‐based subunit coloring scheme and added interactive views with Jmol and occasional watercolor paintings to show cellular context. The illustrative style has been adopted by several publicly available molecular visualization programs and the program used to create the illustrations is freely available as a stand‐alone Fortran program and through a web interface (https://ccsb.scripps.edu/illustrate ^9^).

Echoing our commitment to free and open access of PDB data, the Molecule of the Month was created for community use and reuse. Illustrations from the column are freely available and have been incorporated into student theses, used in classrooms, and appeared on the covers of journals, including American Scientist, Wing Beats (Florida Mosquito Control Association), Zygote Quarterly (science and design), and Transfusion (transfusion medicine). The illustrations are also frequently reprinted in textbooks. Molecule of the Month images have been recognized by several awards and publications, including the 2017 NSF/Popular Science Vizzies (for “Zika Virus”), the 2016 Wellcome Trust Image Awards (for “Ebola Virus Proteins”), and the 2015 FASEB BioArt Awards (for “Ebola Virus Proteins”). Article content forms the basis of the protein modeling events at the Science Olympiad for high schools. Articles are also translated into Japanese and distributed by Protein Data Bank Japan (https://pdbj.org).

As the number of articles grew, related resources have been developed that expand the reach of both the RCSB PDB and the Molecule of the Month series. An iconic poster, called “Molecular Machinery,” featured 75 select PDB structures relative in size and described their critical roles in the functions of living cells. Printed copies were distributed to thousands of students and teachers. Illustrations and text from the series formed the basis of an internationally traveling exhibition called “The Art of Science.” These materials and many others are currently available for no cost to download at PDB‐101 (http://pdb101.rcsb.org), the education portal of the RCSB PDB.^10^

As the corpus of articles grew, we realized that the presentation as a simple chronological list missed an opportunity to provide a biologically meaningful and contextual view of the PDB archive. Consequently, the RCSB PDB developed a web browser to organize these features into high‐level categories about protein synthesis, enzymes, health disease, biological energy, infrastructure, communication, biotechnology, and nanotechnology. This Molecule of the Month browser led to the development of the PDB‐101 website, which has been expanded with many additional materials, including full lesson plans, activities such as paper models, and a variety of posters and interactive explorations.

## 2 WORKING WITH THE MOLECULE OF THE MONTH COMMUNITY

We have been continuously influenced by the needs of our readers. Multiple user surveys and interactions over the years have informed feature development as well as the topics that are highlighted. Comments and suggestions sent to the help desk at info@rcsb.org and web access analytics are regularly monitored.

For example, user feedback informed a redesign of the PDB‐101 website in 2015, with the goal of enhancing access to content related to the Molecule of the Month. Several overarching issues were identified in survey responses, including difficulty in finding materials and the scattered nature of the Molecule of the Month and other PDB‐101 materials. The new site incorporated several design features to address these challenges. First, PDB‐101 currently provides multiple ways to access materials. At the simplest level, a keyword search box was added that displays relevant materials for queries such as “DNA” or “cancer.” The user‐friendly subject browser described above was also created, providing access to materials for common subject categories, similar to the chapters that visitors might find in a textbook.

More recently, we performed an online survey during Spring 2019 to assess how Molecule of the Month features are utilized. Out of 339 responses, ~50% use the column for teaching, ~60% work at a college/university, and ~78% work in biology and biochemistry. Many respondents additionally indicated that they will access Molecule of the Month articles when preparing for teaching. A total of 85% of respondents have utilized one or more of special features of the series, including downloading an image for reuse; playing with 3D interactive view; clicking on the PDB structure ID for more information; consulting references listed; and considering the “Topics for Further Discussion” that are provided with each column. Respondents expressed that Molecule of the Month helps them understand topics in categories such as biomolecular structure and function, health and disease, current advances in structural biology, but also in molecules and the environment, drugs and the brain, genetic mechanisms, bioenergetics, nanotechnology, and bionanotechnology.

**FIGURE 2:**
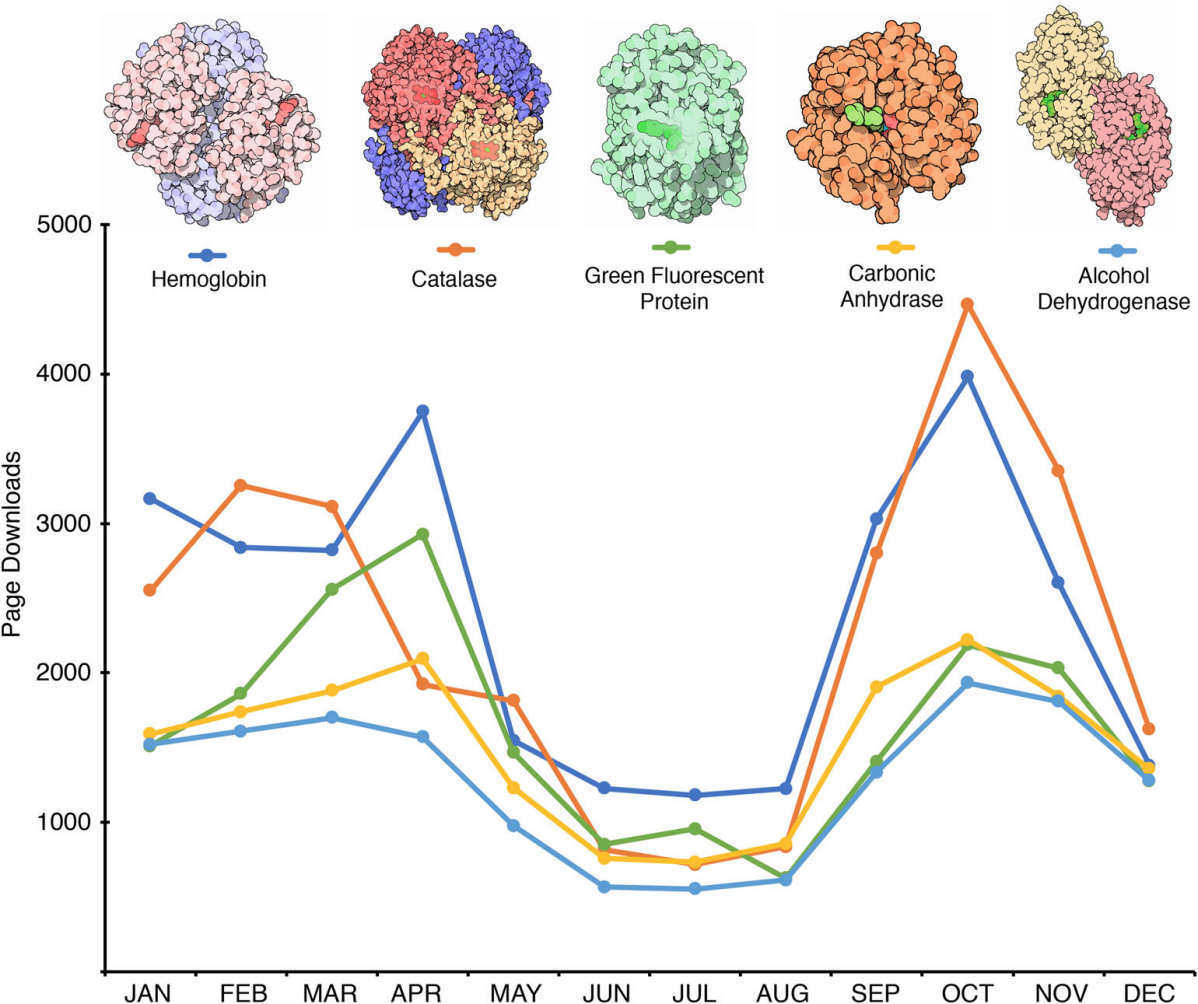
Monthly page downloads in 2018 of the five articles with highest traffic, showing increased activity in months where most classrooms are in session [Color figure can be viewed at wileyonlinelibrary.com]

The PDB‐101 website is visited by more than 600,000 users annually, with Molecule of the Month content representing the majority of pages accessed (64% in 2018). Overall, web traffic declines at the end of the calendar year and during July through August, as might be expected for an educational resource (Figure 2). When looking at traffic for individual articles, two patterns appear. Each new feature is highlighted prominently at the RCSB PDB and PDB‐101 websites, so a particular article will typically show a large spike of traffic for the month of release, then fall to a modest baseline rate. Articles published in 2018 received an average of 10,000 views/year. A subset of entries, however, shows consistent and enduring rates of access. Looking at the topics, the reason for this becomes obvious, with hemoglobin (>28,000 views/year), catalase (>27,000 views/year), green fluorescent protein, and carbonic anhydrase, the most accessed articles. These topics are widely used as examples in classrooms, so there is an ongoing need for such explanatory materials.

## 3 LESSONS LEARNED

December 2019 marked the 20‐year anniversary of the column. These 240 columns have presented an experience for development, progressively improving the content and style of presentations. We have learned a few lessons based on our feedback from visitors.

One of our overarching goals has always been to create materials that minimize jargon and make them accessible to the widest possible audience. This includes both technical language and visual jargon. Scientific language is full of jargon, which is designed to provide a compact and specific way of presenting concepts and ideas. In most cases, this can be translated into natural language by simply using more common terms and allowing a bit more text on the page. In addition, scientific jargon also often contains terms that get entrenched from early work and may be misleading when used later. For example, the term “residue” is often used to denote amino acids and is a term left over from the way that amino acids were isolated from proteins in very early work. Taken alone, this term is misleading, so we prefer to write “an acidic amino acid” rather than “an acidic residue.”

Scientific imagery is also full of jargon, which is easily comprehensible by structural biologists, but looks like a jumble of lines and arrows to people just getting started with science. Our approach has been to create simplified pictures that show the overall shape and form of the molecule, and then provide links to the structural entries to allow more detailed exploration with the interactive viewers available at the RCSB PDB site.

The subjects presented by the column are chosen with a wide range of goals: we want to bring in new visitors and engage our existing community of users. Topics include subjects that are currently in the news, such as the spate of viral outbreaks of Zika, Ebola, and measles, to help users gain understanding of the current level of molecular knowledge related to the topic. Other topics relate to the culture of science, including features that highlight work by winners of the Nobel Prize and articles that highlight new experimental advances like time‐resolved crystallography with X‐ray free‐electron lasers and cryoelectron tomography. Articles are authored to support the PDB‐101 yearly focus on important biomedical topics, such as HIV, diabetes, anti‐bacterial drug resistance, and soon, neurobiology and drug addiction. Other topics are chosen because they relate in familiar ways to our lives and our welfare, including specific topics in biomedicine, biotechnology, and the environment.

One repeated suggestion from our surveys, and from educators in particular, is the need to address larger integrated topics, such as the entire central dogma of genetic mechanisms. The yearly focus was initiated with this goal in mind, to explore biomedical topics in more detail. Going forward, we also plan to explore basic science topics in a similar way, including bioenergetics and foundational genetic mechanisms.

### 3.1 Lessons given

**FIGURE 3:**
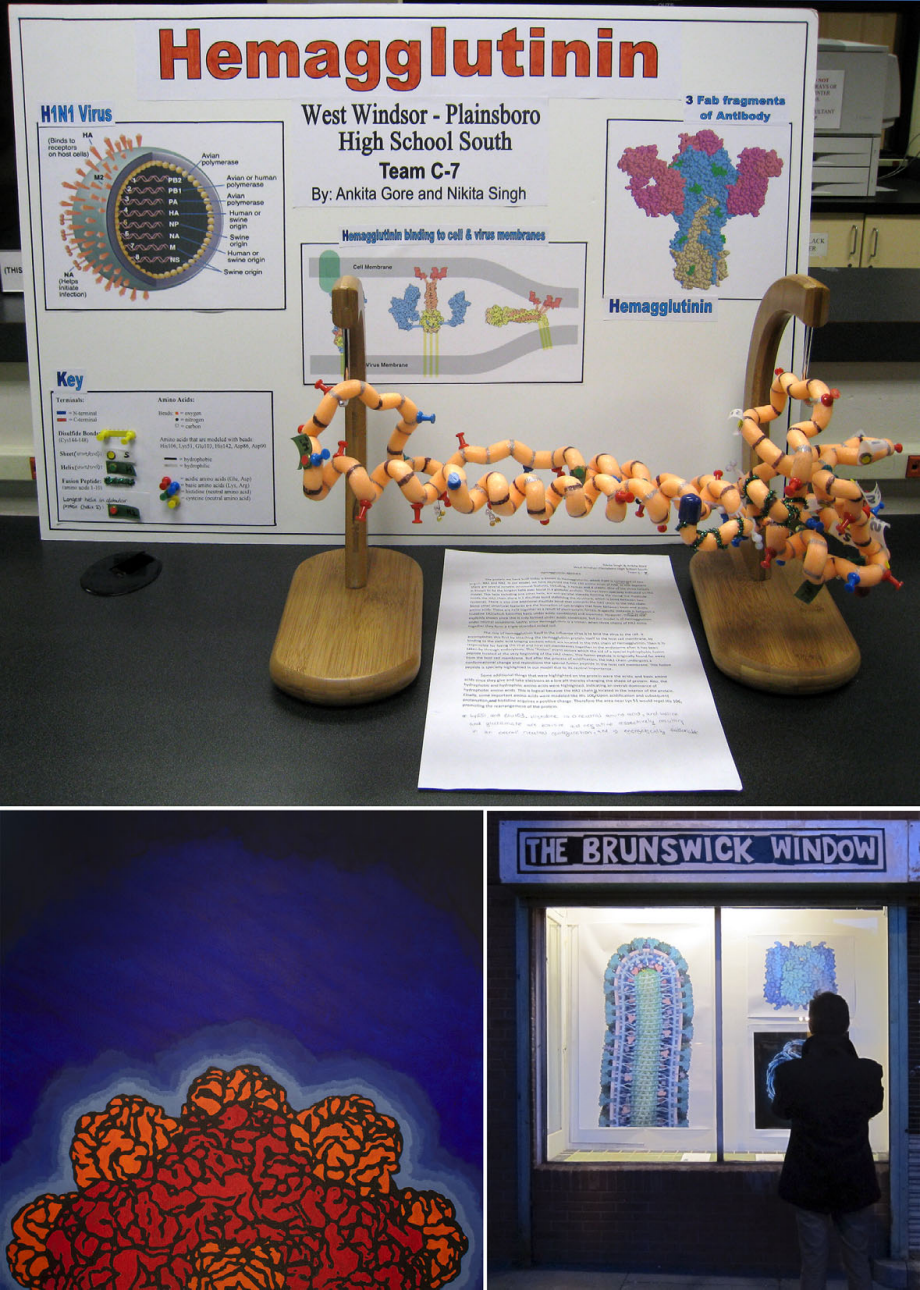
A few uses of the Molecule of the Month. Top: Molecule of the Month images and text used as a resource for the Science Olympiad. Bottom left: painting of bacteriophage phiX174, used as the cover of a book of biomolecular paintings by students at High Tech High.^1^ Bottom right: Molecule of the Month images featured in a public display space used for science/art outreach [Color figure can be viewed at wileyonlinelibrary.com]

The Molecule of the Month has been used in a variety of ways in science education and outreach (Figure 3). As part of the 2019 survey, we asked educators for specific examples of Molecule of the Month PDB‐101 usage in their teaching. Several educators described a process where text and/or illustrations from the Molecule of the Month were used to build a short narrative describing a biological process, such as myosin action or synaptic transmission. As part of testing, students were given these narratives to read, and then asked questions to assess comprehension, and the ability to formulate new hypotheses about the subject. For example, Theresa Diamond at Upper Darby High School mentioned that her younger students struggle to visualize molecules, and this type of imagery can help.

Additional anecdotal examples were solicited through social media, with five responses. In these classrooms, which ranged from high school biology courses to lower division undergraduate courses, educators used the columns to provide an introduction to a given topic, which was then followed by a hands‐on molecular graphics or modeling building activity. Often, the students were allowed to choose topics from the Molecule of the Month archive. Responses from these educators are given in Table 1.

**TABLE 1:** Anecdotal examples of classroom uses

I teach the introductory biochemistry lab to 3rd year students. We try to integrate biochemistry with technology, and I also like to throw some art in there as well. At the end of the semester, the last project we do involves using MotM, UCSF Chimera, and a 3D printer. I have the students work in pairs to choose their favorite biomolecule and learn more about it from the MotM website. Then, they write several questions (about 2–3 pages) pertaining to that molecule. They pair up with another group and swap worksheets, filling in each other's answers. In the end, the students present their molecules, explaining why they are the most interesting. Everyone votes on the best molecule, and the winner gets to 3D print theirs, paint it to highlight the important features, and we use it in lecture the next semester. I'm very proud of the models we have made so far. (Elizabeth Migocovsky, Lecturer in Chemistry Department, San Jose State University)
I use MOM in my Cell Biology class where I have students go on a structure data base scavage hunt. We start at MOM and then they pick a structure and learn how to download it into Chimera and do some basic modeling. (Natalia Hubbs, Asst. Prof. of Biology, Hanover College)
I had my students present proteins they thought were worthy of our protein modeling projects for the ASBMB. They presented, they ranked their favorite winnowing to 5, then voted for favorite. We're studying Apaf‐1 & p53. (Eric Kessler, Director CAPS Bioscience Program)
We read/examine (links) from MOM for topics from ATP synthase through Rubisco and many things in between. Your MOM articles provide an accessible way to understand molecular machines beyond the cartoons of text books. We also use the MOM to build 3D models of the protein, active site or a representative region. Students hold and examine the protein to help understand what was written & how shape = function. Sometimes with foam toobers, large pipe cleaners or by downloading the .pdb files and 3D printing them. With a little editing in Protein visualization software like Jmol we can highlight areas of interest from the MOM articles. Students see more with the models plus the article. (Daniel Williams, teaches HS Biology and Research to Grades 9–12)
After introducing proteins and the fundamentals of protein structure, students read the MOM entry for GFP or insulin before then building the paper models from PDB‐101. We do this in both my non‐majors biology and the majors Cell and Molecular course. I'll also often use the MOM entry as a launching point to show students where to find information on a protein of interest and to find PDB IDs for structures I'd like to 3D print. (Brian J. Gadd, Asst. Prof. of Life Sciences, Los Angeles Mission College)

Andrew Vinal at Wake Technical Community College in Raleigh, NC, has been exploring an ambitious educational application of Molecule of the Month materials. He has partnered with his school's 3D printing facilities and is working with students to create a database of STL files for 3D printing of the molecules included in the column. Students perform the necessary file manipulations to create a 3D printable model using entries from the Molecule of the Month, and they author an enhanced technical story about the molecule under their START project (Student Research Program). The results are made available through a 3D printable model collection on their Model3DBiology website (http://model3dbiology.com) currently under development.

### 3.2 Future of the biostructural education and outreach

One recurring question that we get whenever discussing PDB‐101 and the Molecule of the Month is: “Aren't you running out of molecules?” We are happy to report that this is not even close to true. The PDB archive continues to grow exponentially, recently surpassing 150,000 structural entries. New biostructural methodologies, such as cryoelectron microscopy and time‐resolved crystallography with X‐ray free‐electron lasers, are opening up entirely new fields of structural study and providing structures of ever‐increasing complexity for inclusion in the column. In response to comments from the education community, we are also expanding our thematic offerings, combining the Molecule of the Month with additional materials to explore large topics such as “Bacterial Drug Resistance,” and in the next few years, “Drugs and the Brain” and “Cancer and Genetics.” Through the Molecule of the Month, we will continue to entice new visitors to explore structural biology, and in association with PDB‐101, support the educational community with friendly resources for exploring biomolecular structure and function.
